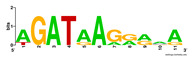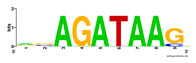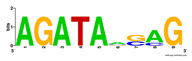# A Clustering Approach for Motif Discovery in ChIP-Seq Dataset

**DOI:** 10.3390/e21080802

**Published:** 2019-08-16

**Authors:** Chun-xiao Sun, Yu Yang, Hua Wang, Wen-hu Wang

**Affiliations:** 1 College of Science, Northwest A&F University, Yangling 712100, China; 2 School of Computer Science, Pingdingshan University, Pingdingshan 467000, China; 3 School of Mathematical Sciences, Shanghai Jiao Tong University, Shanghai 200240, China; 4 College of Software, Nankai University, Tianjin 300071, China; 5 Department of Mathematical Sciences, Georgia Southern University, Statesboro, GA 30460, USA

**Keywords:** motif discovery, transcription factor binding sites, planted motif search, ChIP-Seq

## Abstract

Chromatin immunoprecipitation combined with next-generation sequencing (ChIP-Seq) technology has enabled the identification of transcription factor binding sites (TFBSs) on a genome-wide scale. To effectively and efficiently discover TFBSs in the thousand or more DNA sequences generated by a ChIP-Seq data set, we propose a new algorithm named AP-ChIP. First, we set two thresholds based on probabilistic analysis to construct and further filter the cluster subsets. Then, we use Affinity Propagation (AP) clustering on the candidate cluster subsets to find the potential motifs. Experimental results on simulated data show that the AP-ChIP algorithm is able to make an almost accurate prediction of TFBSs in a reasonable time. Also, the validity of the AP-ChIP algorithm is tested on a real ChIP-Seq data set.

## 1. Introduction

Transcription factor binding sites (TFBSs) [1] are short, degenerated nucleotide fragments (usually ≤30 bps) located in specific DNA regions. They play an important role in regulating gene expression. The Planted (l,d) Motif Search (PMS) problem [2] is a popular motif model for the identification of TFBSs (i.e., motif discovery) in bioinformatics, and is formally defined as follows:

**Definition** **1** (PMS)**.** *Given a set of DNA sequences $X=\{X_{1},X_{2},\cdots ,X_{t}\}$ with $|X_{i}|=n$ and three non-negative integers d,q,l, with 0≤d<l<n,0<q≤t, the PMS problem is to find an l-mer M (a string of length l), such that each selected sequence $X_{i}$ has an l-mer $M_{i}$ with Hamming distance $d_{H}\left(M,M_{i}\right)\leq d$, for i=1,2,⋯,q. The l-mer M is called an (l,d) motif and the l-mer $M_{i}$ is called a motif instance.*

According to the different values of *q* representing the distribution of motif instances, there are three different motif discovery sequence models [3]: (i) Exactly one motif occurrence per sequence (the OOPS model), (ii) zero or one motif occurrences per sequence (the ZOOPS model), or (iii) zero or more motif occurrences per sequence (the TCM model). For the OOPS model, q=t, for the ZOOPS model and TCM model, 0<q<t.

Generally, there are two kinds of algorithms for solving the PMS problem: exact algorithms and approximate algorithms. Exact algorithms [4,5,6,7,8,9,10] always use consensus sequences [11] to represent motifs and can find all (l,d) motifs. Most exact algorithms are pattern-driven algorithms, which attempt to enumerate all possible $4^{l}$*l*-mers (substring patterns of length *l*) to find the *l*-mer with the maximum number of approximate occurrences. Approximate algorithms [12,13,14,15,16,17,18] usually use a position weight matrix (PWM) [19] to describe the most likely occurring motifs and can report results in a short time, but do not always identify all (l,d) motifs. Most approximate algorithms use probabilistic analysis to maximize the score function which describes how likely it is for an *l*-mer pattern to be a motif instance.

Recently, chromatin immunoprecipitation combined with next-generation sequencing (ChIP-Seq) technology has produced extremely valuable information for the genome-wide identification of transcription factor binding sites (TFBSs) and in the field of epigenetics, which mainly focus on DNA methylation, nucleosome localization, and histone modification. For transcription factors, ChIP-Seq is widely used to study the binding of transcription factors for the analysis of gene expression regulation on a genome-wide scale. For histones, ChIP-Seq performs high-throughput histone modification sequencing in the whole genome with sufficient sequencing depth and range, which not only improves the sensitivity and specificity of sequencing, but can also transform qualitative sequencing methods into quantitative detection.

In this paper, we focus on an algorithm to discover transcription factor binding sites in a ChIP-Seq data set. A ChIP-Seq data set is a set of peak regions containing TFBSs obtained through ChIP-Seq experiments, read mapping, and peak calling. It contains hundreds or more DNA sequences, which increases the difficulty of accurate and efficient identification of TFBSs.

Some algorithms have recently been proposed to discover TFBSs in ChIP-Seq data sets [20,21,22,23,24,25,26,27,28,29]. However, none of them has proven to be absolutely superior, compared to the rest. Some of these are tailored versions of previous motif discovery algorithms, specifically tailored towards ChIP-Seq data sets, such as MEME-ChIP [20] and HMS [21]. MEME-ChIP [20], which incorporates two complementary motif discovery algorithms, known as MEME and DREME [22], can identify motifs without restriction on the size or number of sequences, allowing very large ChIP-Seq data sets to be analyzed. HMS [21], which is an improved version of Gibbs Sampler, combines stochastic sampling and a deterministic greedy search step, which improves computation efficiency. DREME [22] is specifically designed to find short, core DNA-binding motifs of eukaryotic TFs, and is optimized to analyze very large ChIP-Seq data sets in just minutes. One may speed up the existing motif discovery algorithms by integrating some information, such as in the cases of STEME [23] and ChIP-Munk [24]. STEME [23] accelerates MEME by indexing sequences with suffix trees. ChIP-Munk [24] combines a greedy approach with an expectation-maximization (EM) algorithm to achieve a high efficiency. There are also exhaustive methods for determining exact motifs in ChIP-Seq data sets, such as FMotif [25] and Weeder [26]. FMotif [25] first constructs a mismatched suffix tree to scan and count all possible motif instances, and then implements a depth-first search to enumerate all possible motifs. However, the run time of FMotif becomes unrealistic with increasing values of *l* and *d*. Others use word enumeration methods to process full-size ChIP-Seq data sets, such as CisFinder [28] and MCES [29]. CisFinder [28] employs a word clustering method to group short *l*-mers (l=7, 8, or 9), but struggles to find exact (l,d) motifs with larger values of *l* and *d* in ChIP-Seq data sets. MCES [29], a new planted (l,d) motif discovery algorithm, mines and combines substrings to rapidly identify exact motifs in full-size ChIP-Seq data sets.

In this paper, we propose a new motif discovery algorithm, named AP-ChIP, which is specially designed for better discovering TFBSs in ChIP-Seq data sets. The algorithm first constructs and then further filters cluster subsets using probabilistic analysis. Then, Affinity Propagation (AP) clustering [30] is applied to the candidate cluster subsets in order to discover optimal motifs. Experimental results show that the AP-ChIP Algorithm 1 can find TFBSs in a ChIP-Seq data set very efficiently and effectively.

## 2. Method

A ChIP-Seq data set has the following fundamental features: (i) Some of the sequences may contain no motifs at all; and (ii) thousands of sequences lead to huge amount of background *l*-mers. To cater to ChIP-Seq data sets, we design the AP-ChIP Algorithm 1 under the ZOOPS model and set some proper thresholds to filter redundant background *l*-mers. More specifically, the AP-ChIP Algorithm 1 consists of the following three steps:

### 2.1. Construct Cluster Subsets

We introduce the observation that any two motif instances $x_{1}$ and $x_{2}$, each of which differs from the same motif *x* up to *d* positions, must have Hamming distance of no more than 2d, denoted as $d_{H}\left(x_{1},x_{2}\right)\leq 2d$. Consequently, if an *l*-mer in one sequence is a motif instance, all other motif instances in the remaining sequences will be gathered in the corresponding cluster subset. Under the ZOOPS model, we choose h(h=t−q+1) sequences as the reference sequences to ensure that at least one sequence among the *h* sequences contains a motif instance [31]. In general, we use the first *h* sequences $\left\{X_{1},X_{2},\cdots ,X_{h}\right\}$ as the reference sequences. As the *l*-mer which is the motif instance is not known in advance, we consider all *l*-mers $x_{i,j}$(i=1,2,⋯,h;j=1,2,⋯,n−l+1) in the first *h* sequences as the reference subsequences.

The ideal cluster subsets are expected to contain as few background *l*-mers as possible and, also, to include sufficient motif instances. Therefore, we set a threshold *k*(d<k≤2d), so that, for each reference subsequence $x_{i,j}$, all *l*-mers $x_{i^{\prime },j}$$(i^{\prime }=1,2,\cdots ,t$, $i^{\prime }\neq i;j=1,2,\cdots ,n-l+1)$ in the whole sequences, except the *i*th sequence $X_{i}$ such that $d_{H}\left(x_{i,j},x_{i^{\prime },j}\right)\leq k$, are selected to construct a cluster subset; that is

(1) $$\begin{array}{c} C\left(x_{i,j},X\right)=\left\{x_{i,j}\right\}\overset{t}{\underset{i^{\prime }=1\wedge i^{\prime }\neq i}{⋃}}B\left(x_{i,j},X_{i^{\prime }}\right), \end{array}$$

where $B\left(x_{i,j},X_{i^{\prime }}\right)=\left\{x_{i^{\prime },j}:x_{i^{\prime },j}\in_{l}X_{i^{\prime }},d_{H}\left(x_{i,j},x_{i^{\prime },j}\right)\leq k\right\}$ represents the set of the selected *l*-mers in the $i^{\prime }$th sequence $X_{i^{\prime }}$ and $x_{i^{\prime },j}\in_{l}X_{i^{\prime }}$ if and only if $x_{i^{\prime },j}$ is an l−mer of the sequence $X_{i^{\prime }}$.

To set a proper threshold *k*, two probabilistic expressions are employed. The first is the probability of the Hamming distance between two random *l*-mers $x_{1}$ and $x_{2}$ being no more than *k* [4]:

(2) pk=p(dH(x1,x2)≤k)=∑i=0kli34i14l−i.

The other is the probability of the Hamming distance between two selected motif instances $m_{1}$ and $m_{2}$ being no more than *k*: [18].

(3) $$\begin{array}{c} p_{kmotif}=p\left(d_{H}\left(m_{1},m_{2}\right)\leq k\right). \end{array}$$

Now, we describe the method for calculating $p_{kmotif}$. Given two motif instances, $m_{1}$ and $m_{2}$, of the same motif $m_{0}$ with up to *d* mutations, the distances between $m_{1},$$m_{2}$, and $m_{0}$ satisfy $d_{H}\left(m_{0},m_{1}\right)=\alpha$ and $d_{H}\left(m_{0},m_{2}\right)=\beta$, (0≤α≤d,0≤β≤d). Thus, the probability $p_{kmotif}$ can be calculated as

(4) $$\begin{array}{ll} p_{kmotif} & =p(d_{H}\left(m_{1},m_{2}\right)\leq k)\\ & =p\left(d_{H}\left(m_{1},m_{2}\right)\leq k|d_{H}\left(m_{0},m_{1}\right)=\alpha ,d_{H}\left(m_{0},m_{2}\right)=\beta \right)\times p\left(\alpha ,\beta \right), \end{array}$$

where $p(d_{H}\left(m_{1},m_{2}\right)\leq k|d_{H}\left(m_{0},m_{1}\right)=\alpha ,d_{H}\left(m_{0},m_{2}\right)=\beta )$ represents the conditional probability of $p(d_{H}\left(m_{1},m_{2}\right)\leq k)$ given $d_{H}\left(m_{0},m_{1}\right)=\alpha$, $d_{H}\left(m_{0},m_{2}\right)=\beta$, such that

(5) p(dH(m1,m2)≤k|dH(m0,m1)=α,dH(m0,m2)=β)=∑i=[α+β−k2]+1min(α,β)αi×l−αβ−i×3βlβ×3βk<α+β≤2d,10<α+β≤k,

and p(α,β) represents the probability of $d_{H}\left(m_{0},m_{1}\right)=\alpha$ and $d_{H}\left(m_{0},m_{2}\right)=\beta$; that is

(6) $$\begin{array}{c} p\left(\alpha ,\beta \right)=p(d_{H}\left(m_{0},m_{1}\right)=\alpha ,d_{H}\left(m_{0},m_{2}\right)=\beta ). \end{array}$$

As $d_{H}\left(m_{0},m_{1}\right)=\alpha$ and $d_{H}\left(m_{0},m_{2}\right)=\beta$ are independent, we have

(7) $$\begin{array}{c} p\left(\alpha ,\beta \right)=p\left(d_{H}\left(m_{0},m_{1}\right)=\alpha \right)\times \left(d_{H}\left(m_{0},m_{2}\right)=\beta \right), \end{array}$$

(8) p(dH(m0,m1)=α)=dα3α4d,

(9) p(dH(m0,m2)=α)=dβ3β4d.

Combining Equations (5)–(9), we have

(10) pkmotif=p(dH(m1,m2)≤k),=∑0≤α,β≤d∑i=[α+β−k2]+1min(α,β)αil−αβ−i×3βlβ×3β×2dα3α42d×2dβ3β42dk<α+β≤2d,(∑0≤α,β≤d1)×2dα3α42d×2dβ3β42d0<α+β≤k.

Having calculated the two probabilities $p_{k}$ and $p_{kmotif}$, we now describe how to set the proper threshold *k*, in order to construct the cluster subsets which contain as few background *l*-mers as possible while including sufficient motif instances. A larger value of $p_{kmotif}$ indicates more motif instances belong to the cluster subset; however, a smaller value of $p_{k}$ suggests that fewer background *l*-mers appear in the same cluster subset. Therefore, the threshold *k* should be set in a way that ensures that the value of $p_{kmotif}$ is large enough, compared to the value of $p_{k}$.

To demonstrate this issue, let us consider the (18, 5) problem instance as an example. The values of $p_{k}$ and $p_{kmotif}$ are shown in Table 1. When k=7, the value of $p_{kmotif}$ is 0.7414, which allows us to obtain sufficient motif instances, whereas the value of $p_{k}$ is 0.0012 which, in turn, allows us to reduce the scale of background *l*-mers in the same cluster subset. Therefore, the optimal value of *k* is 7.

### 2.2. Filter Cluster Subsets

As is known, the true motif instances must exist in one of these h×(n−l+1) cluster subsets $C(x_{i,j},X)$, which are constructed with the reference subsequences $x_{i,j}$(i=1,2,⋯,h;j=1,2,⋯,n−l+1) from the first *h* sequences. However, with a great number of total cluster subsets, the identification of the cluster subsets that contain the true motif instances is highly time-consuming as most of these cluster subsets have redundant background *l*-mers.

To filter the interference cluster subsets, a threshold $p_{occ}^{f}$ (i.e., an occurrence frequency) [29] is employed with the purpose of analyzing the probability of a random motif instance $x^{\prime }$ occurring in a given sequence.

(11) poccf=∑i=0ddi×pmuti×(1−pmuti)d−i×1li×3i,

where $p_{mut}$ is the probability of a character mutation in one position. For each sequence $X_{i^{\prime }}\left(i^{\prime }=1,2,\cdots ,t,i^{\prime }\neq i\right)$, if the number of the selected *l*-mers in $B(x_{i,j},X_{i^{\prime }})$, denoted as $\left|B(x_{i,j},X_{i^{\prime }})\right|$, is greater than or equal to $p_{occ}^{f}\times \left(n-l+1\right)$,

(12) $$\begin{array}{c} |B\left(x_{i,j},X_{i^{\prime }}\right)|\geq p_{occ}^{f}\times \left(n-l+1\right), \end{array}$$

then the sequence $X_{i^{\prime }}$ may contain a motif instance and is stored in a set N(i,j). For each N(i,j), if the number of the selected sequences in N(i,j), denoted as |N(i,j)|, is not less than *q*,

(13) $$\begin{array}{c} |N(i,j)|\geq q, \end{array}$$

then there are at least *q* possible motif instances in the corresponding cluster subset $C(x_{i,j},X)$, the reference subsequence $x_{i,j}$ is considered as a potential motif instance, and the corresponding cluster subset $C(x_{i,j},X)$ is a candidate cluster subset $C_{candidate}\left(x_{i,j},X\right)$.

### 2.3. Refine Cluster Subsets

Due to the occurrence frequency $p_{occ}^{f}$ and the relatively small Hamming distance *k* between the reference subsequence and the selected *l*-mers, only a limited number of cluster subsets, which contains a small number of the selected *l*-mers, are retained as candidate cluster subsets for further Affinity Propagation (AP) clustering. In order to quickly produce highly conserved cluster subsets, we apply AP clustering to each candidate cluster subset. Compared to other clustering approaches, AP clustering can cluster large-scale data sets efficiently by exchanging messages between data points.

#### 2.3.1. Affinity Propagation (AP) Clustering

As demonstrated in our recent work [32], it is possible to speed up AP clustering and improve its accuracy with the adapted similarity s(i,k), which is based on pair-wise constraints and a variable-similarity measure [33]. The adapted similarity, s(i,k), between two *l*-mers $x_{i,j}$ and $x_{k,j}$ is defined as

(14) $$\begin{array}{c} s\left(i,k\right)=-\rho \times d_{H}\left(x_{i,j},x_{k,j}\right)\times L\left(x_{i,j},x_{k,j},X\right), \end{array}$$

where

(15) $$\begin{array}{c} \rho =\left\{\begin{array}{cc} R_{1} & \mathrm{if}\;d_{H}\left(x_{i,j},x_{k,j}\right)\in \left(0,k\right]\\ R_{2} & \mathrm{if}\;d_{H}\left(x_{i,j},x_{k,j}\right)\in \left(k,2k\right]\\ +\infty & \mathrm{if}\;d_{H}\left(x_{i,j},x_{k,j}\right)\in \left(2k,4k\right] \end{array}\right. \end{array}$$

(16) $$\begin{array}{c} L\left(x_{i,j},x_{k,j},X\right)=\left\{\begin{array}{cc} +\infty & \mathrm{if}\;x_{i,j}\in_{l}X_{p},x_{k,j}\in_{l}X_{q},p=q\\ 1 & \mathrm{otherwise}\; \end{array}\right.. \end{array}$$

Note that $R_{1}\in \left(1,+\infty \right)$ and $R_{2}\in \left(0,1\right]$.

For each candidate cluster subset, based on the adapted similarity s(i,k), AP clustering recursively calculates two types of messages. The first type is the responsibility r(i,k), which reflects the suitability of point $x_{k,j}$ as the exemplar for point $x_{i,j}$. The other type is the availability a(i,k), which indicates how suitable it would be for a point $x_{i,j}$ to choose the point $x_{k,j}$ as its exemplar:

(17) $$\begin{array}{c} r\left(i,k\right)=s\left(i,k\right)-\max_{x_{k^{\prime },j}\neq x_{k,j}}\left\{a\left(i,k^{\prime }\right)+s\left(i,k^{\prime }\right)\right\}, \end{array}$$

(18) $$\begin{array}{c} a\left(i,k\right)=min\left\{0,r\left(k,k\right)+\sum_{x_{i^{\prime },j}\neq \left\{x_{i,j},x_{k,j}\right\}}max\left\{0,r\left(i^{\prime },k\right)\right\}\right\}\;\mathrm{if}\;x_{i,j}\neq x_{k,j}, \end{array}$$

(19) $$\begin{array}{c} a\left(k,k\right)=\sum_{x_{i^{\prime },j}\neq x_{k,j}}max\left\{0,r\left(i^{\prime },k\right)\right\}\}. \end{array}$$

When the AP clustering converges, a set of *l*-mers in the produced cluster subset are selected as exemplars e(i) associated to the point $x_{i,j}$:

(20) $$\begin{array}{c} e\left(i\right)=\underset{\;\;x_{k,j}}{arg max}\left\{r\left(i,k\right)+a\left(i,k\right)\right\}. \end{array}$$

#### 2.3.2. Cluster Subset Refinement

To select an adequate number of desired cluster subsets with more motif instances but less background *l*-mers, we set an interval [min size, max size] = [t−q,t] to further refine the cluster subsets $C_{AP}\left(x_{i,j},X\right)$, which is produced, by AP clustering, using a reference *l*-mer $x_{i,j}$. Regarding the number of the *l*-mers in each cluster subset $C_{AP}\left(x_{i,j},X\right)$, we conclude that there are three cases:

(i) In the case where the number is less than t−q (for example, $|C_{AP}\left(x_{i,j},X\right)\left|<t-q\right)$, such a small number of *l*-mers is not enough to create a cluster subset $C_{AP}\left(x_{i,j},X\right)$ which represents a true motif, so we consider it as an invalid cluster subset.

(ii) In the case where the number is between t−q and *t* (for example $t-q<|C_{AP}\left(x_{i,j},X\right)\left|\leq t\right)$, we consider it as a valid cluster subset.

(iii) In the case where the number is more than *t* (for example $|C_{AP}\left(x_{i,j},X\right)|>t$), the size of the cluster subset is so large that it may include too many background *l*-mers and, so, we use a greedy strategy to select *tl*-mers from $C_{AP}\left(x_{i,j},X\right)$ to form $C_{valid}\left(x_{i,j},X\right)$. First, $C_{valid}\left(x_{i,j},X\right)$ is initialized with the AP clustering exemplar e(i). Then, an *l*-mer $x_{r,j}$ from $C_{AP}\left(x_{i,j},X\right)-C_{valid}\left(x_{i,j},X\right)$ is repeatedly chosen, following Equations (21) and (22), and added to $C_{valid}\left(x_{i,j},X\right)$ until $|C_{valid}\left(x_{i,j},X\right)\left|=t\right)$:

(21) $$\begin{array}{c} x_{r,j}=\underset{x_{i,j}\in C_{AP}-C_{valid}}{arg max}\sum_{x_{k,j}\in C_{valid}}sim\left(x_{i,j},x_{k,j}\right), \end{array}$$

(22) $$\begin{array}{c} sim\left(x_{i,j},x_{k,j}\right)=\frac{len(x_{i,j},x_{k,j})}{|x_{i,j}\left|+\right|x_{k,j}|-len\left(x_{i,j},x_{k,j}\right)}, \end{array}$$

where $len(x_{i,j},x_{k,j})$ is the length of the maximum intersection of $x_{i,j}$ and $x_{k,j}$.

To appropriately sort the valid cluster subsets $C_{valid}\left(x_{i,j},X\right)$, we use the information content (IC) [34], and the cluster subset with the maximum value of IC is considered as the true motif model:

(23) $$\begin{array}{c} IC\left(C_{valid}\left(x_{i,j},X\right)\right)=\sum_{m=1}^{l}\sum_{w=1}^{4}p_{w,m}\log\frac{p_{w,m}}{p_{w,0}}, \end{array}$$

where $p_{w,m}$ is the probability of the character w∈A,T,C,G at the position *m* of the *l*-mer $x_{i,j}$, and $p_{w,0}$ is the corresponding background probability.

Based on the above described three steps, the whole AP-ChIP Algorithm 1 is described as follows:

**Algorithm 1:**AP-ChIP algorithm

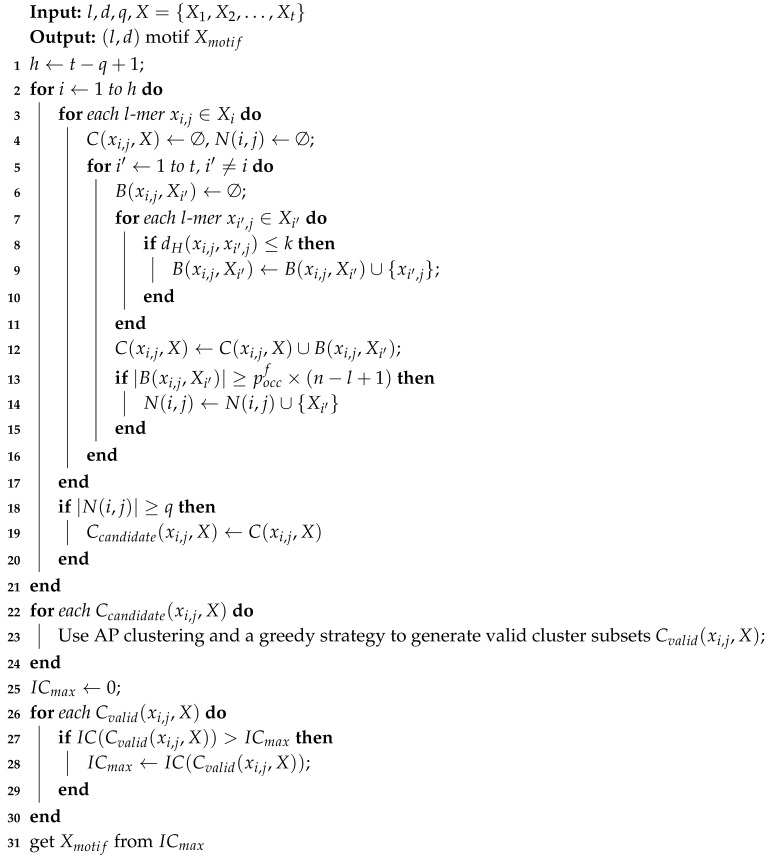



Steps 2–17 describe the process of constructing the cluster subsets. Steps 18–21 describe the filtration of the cluster subsets. Steps 22–31 describe the refinement of the cluster subsets and the verification of the motif with the maximum IC score.

## 3. Results

### 3.1. Results on Simulated Data

Simulated data provide quantitative measures to test the performance of the AP-ChIP Algorithm 1. As in [29], we generate the simulated data as follows:

First, we generated *t* independent and identically distributed (i.i.d) sequences of length *n* and a motif *m* of length *l*. Second, we randomly generated *q*(0<q≤t) motif instances, each of which differed from the motif *m* at up to *d* positions. Third, the *q* motif instances were placed in a random position in a random selection of *q* sequences selected out of the *t* sequences. We, then, implemented the AP-ChIP Algorithm 1, using Matlab on a computer with a 2.6 GHZ processor and 4 Gbyte memory. The final experimental results consisted of the averages of five trials of simulated data experiments.

To evaluate the motif prediction accuracy, the nucleotide level performance coefficient (nPC), as defined by Pevezner and Sze [2], was used:

(24) $$\begin{array}{c} nPC=\frac{\left|K\cap P\right|}{\left|K\cup P\right|}, \end{array}$$

where *K* is the set of nucleotide positions in the true motif and *P* is the corresponding set of nucleotide positions in the predicted motif. The value of nPC is between 0 and 1; the larger the value of nPC, the higher the accuracy of the predicted motif. We used the 2d neighborhood probability $p_{2d}$ [8] to select a group of (l,d) motif instances. The larger the value of $p_{2d}$, the weaker the corresponding (l,d) problem instance becomes:

(25) p2d=P(dH(x1,x2)≤2d)=∑i=02dli34i14l−i.

In what follows, according to different values of $\alpha =\frac{q}{t}$ (i.e., the ratio of the sequences containing motif instances to all the sequences), we designed two groups of experiments, both of which consisted of two sub-experiments, to test the performance of the AP-ChIP Algorithm 1 on simulated data sets.

In the first group of experiments, we set α=100% for both sub-experiments. We compared the performance of the AP-ChIP Algorithm 1 with that of the widely used motif finding algorithms MEME [13], VINE [14], and Projection [16].

In the first sub-experiment, we set the number of sequences as t=20 with sequence length n=600. The running time and the predicted accuracy of these algorithms are shown in Table 2. For the instances (12, 2) and (15, 3) with $p_{2d}<0.05$, the AP-ChIP Algorithm 1 achieved near-optimal predicted accuracy within a relatively short time. For the instances (15, 4), (14, 4), (25, 8), and (21, 7) with $p_{2d}\geq 0.05$, the predicted accuracy of the AP-ChIP Algorithm 1 was over 90% and the running time of the AP-ChIP Algorithm 1 remained competitive, compared to the other algorithms.

In general, it is easy to find the true motif by increasing the sequence number and decreasing its length. Therefore, in the second sub-experiment, we set the sequence number as t=1000 with sequence length n=200. Table 3 shows that, for all (l,d) problem instances with $p_{2d}\geq 0.05$, the predicted accuracy of the AP-ChIP Algorithm 1 is over 90% with the computational costs being satisfactory.

Next, in the second group of experiments, to simulate a real ChIP-Seq data set, we set α=90% for both sub-experiments. This is because in real ChIP-Seq data set, most but not all of the sequences contain motif instances.

In the first sub-experiment, we test the validity of the AP-ChIP Algorithm 1 on the simulated ChIP-Seq data set for the identification of (l,d) motifs with t=1000 and n=200. We choose $p_{2d}=0.05$ to select a group of (l,d) problem instances. The reason for this choice is that $p_{2d}=0.05$ is approximately the same as the $p_{2d}$ value of the (15, 4) problem instance, which is one of the most popular benchmarks for (l,d) problem instance. The running time and the predicted motif by the AP-ChIP Algorithm 1 are shown in Table 4. For each (l,d) problem instance, the AP-ChIP Algorithm 1 finds almost the same motif as the published one and also runs quite efficiently.

In the second sub-experiment, in order to further demonstrate the performance of the AP-ChIP Algorithm 1 on the simulated ChIP-Seq data set, we compared the AP-ChIP Algorithm 1 against some established motif-finding algorithms in the following two aspects: (i) Different values of $p_{2d}$, ranging from 0.05 to 0.5, with fixed α=90%, and (ii) different values of α floating from 0.7 to 1 with fixed (l,d)=(9,2). Although a genome-wide ChIP-Seq data set typically has thousands to tens of thousands of sequences, using 20% to 50% of the ChIP-Seq data set is usually adequate for obtaining a good estimate of the true motifs. MEME-ChIP, a well-known algorithm for discovering motifs in ChIP-Seq data sets, is able to well identify (l,d) motifs with only 600 sequences. Thus, it was reasonable to set the sequence number as t=600 and sequence length as n=200 for motif discovery in our experiments.

First, we compared the running time and prediction accuracy of the AP-ChIP Algorithm 1 with those of the three compared algorithms, MEME-ChIP [20], ChIP-Munk [24], and FMotif [25], on different values of $p_{2d}$ and with fixed α=90%. As shown in Table 5, for each (l,d) problem instance, the AP-ChIP Algorithm 1 could solve it in a relatively short time, and its prediction accuracy was better than those of the three compared algorithms. Specifically, with increasing values of *l* and *d*, FMotif found it difficult to find exact motifs.

**Figure 1:**
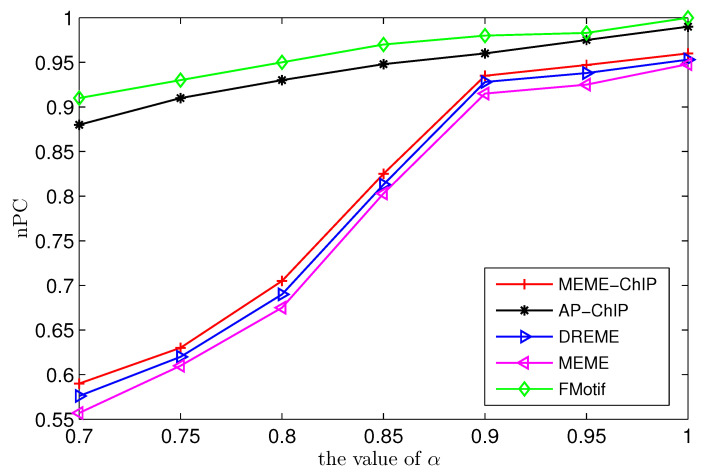
Prediction accuracy for different values of α.

Next, we compared the prediction accuracy of AP-ChIP Algorithm 1 with those of the algorithms MEME [13], MEME-ChIP [20], DREME [22], and FMotif [25], on different values of α floating from 0.7 to 1 and fixed (l,d)=(9,2). As the ratio of a motif instance $\alpha =\frac{q}{t}$ has a strong effect on the prediction accuracy, it was necessary for us to test the prediction accuracy of AP-ChIP Algorithm 1 on different values of α. It is rather cumbersome to identify the true motif when the value of α is small. FMotif is a powerful, exhaustive algorithm for finding exact short (l,d) motifs (l≤10,d≤2) contained in ChIP-Seq data sets. As shown in Figure 1, the prediction accuracy of AP-ChIP Algorithm 1 was nearly the same as that of FMotif, and was higher than that of MEME-ChIP, MEME, and DREME.

### 3.2. Results on Real Data

First, we tested the validity of the AP-ChIP Algorithm 1 for the identification of real motifs using a diverse set of real ChIP-Seq data sets; specifically, on 12 differently sized mESC data sets (Nanog, Oct4, Sox2, Esrrb, Zfx, Klf4, c-Myc, n-Myc, Tcfcp21l, Smad1, STAT3, and CTCF) [35]. We compared the motifs detected by the AP-ChIP Algorithm 1 with the motifs published by Chen et al. [35], and presented them in the form of sequence logos [36], which graphically represent the degree of motif conservation, as measured by relative entropy. Table 6 shows the running times and the predicted motifs of the AP-ChIP Algorithm 1. For each data set, the AP-ChIP Algorithm 1 was capable of finding motifs highly similar to the published ones within a reasonable time.

Moreover, to better show the results, we compared AP-ChIP 1 with MEME-ChIP on nine differently sized ENCODE data sets (Nfyb, Hnf4, Elf1, Ets, Egr1, Yy1, Six5, Srf, and Tal1) [37], where the TFBSs were referenced in the JASPAR database [38]. Table 7 shows the published motifs and the motifs predicted by the two algorithms. The motifs are also shown in the form of sequence logos. AP-ChIP 1 could successfully find a motif similar to the published motif for each data set, while, for some data sets MEME-ChIP failed to accurately predict the motif (e.g., in the Elf1 data set), or lost information on individual bases (e.g., in the Tal dataset).

## 4. Concluding Remarks

In this paper, our goal was to find a method providing balance between time performance and prediction accuracy for TFBS discovery in ChIP-Seq data sets. Consequently, we aimed to obtain these results with high prediction accuracy in a relatively short time. To do so, we proposed a novel clustering-based algorithm named AP-ChIP 1. Firstly, to achieve high prediction accuracy, we set a threshold *k* to restrict the number of the selected *l*-mers in the candidate cluster subsets. Next, to obtain good time performance, we set the threshold $p_{occ}^{f}$ in terms of probabilistic analysis, in order to filter the interferential candidate cluster subsets. Furthermore, a powerful data clustering method, AP clustering, was used to obtain the almost accurate motifs. Experimental results on both simulated and real ChIP-Seq datasets showed that the AP-ChIP Algorithm 1 not only discovers the motifs as consistently as the published ones, but also does so quite efficiently. This demonstrates that the AP-ChIP Algorithm 1 is a powerful new approach for ChIP-Seq data set analysis which provides a good trade-off between time performance and prediction accuracy.

## Figures and Tables

**Table 1:** Values of $p_{k}$ and $p_{kmotif}$ under different values of *k* for (18, 5) problem instance.

*k*	5	6	7	8	9	10
$p_{k}$	$3.42\times 10^{-5}$	$2.31\times 10^{-4}$	0.0012	0.0054	0.0193	0.0569
$p_{kmotif}$	0.2303	0.4741	0.7414	0.9242	0.9915	1

**Table 2:** Comparisons on (l,d) problem instances with t=20, n=600, and α=100%.

(l,d)	$p_{2d}$	MEME	VINE	Projection	AP-ChIP
(12, 2)	0.0028	0.68 (4 s)	1.00 (8 s)	0.86 (10 s)	0.98 (18 s)
(15, 3)	0.0042	0.73 (7 s)	1.00 (9 s)	0.82 (1.3 m)	1.00 (23 s)
(15, 4)	0.0566	0.87 (8 s)	0.96 (5.6 m)	0.89 (4.2 m)	0.97 (36 s)
(14, 4)	0.1117	0.84 (10 s)	0.95 (8.3 m)	0.80 (27.4 m)	0.96 (47 s)
(25, 8)	0.1494	0.91 (12 s)	0.93 (9.8 m)	0.78 (32.6 m)	0.94 (1.1 m)
(21, 7)	0.2564	0.87 (28 s)	0.92 (11.2 m)	0.76 (48.7 m)	0.91 (58 s)

**Table 3:** Comparisons on (l,d) problem instances with t=1000, n=200, α=100%.

(l,d)	$p_{2d}$	MEME	VINE	Projection	AP-ChIP
(12, 3)	0.0540	0.94 (8 s)	0.96 (2.4 m)	0.91 (3.1 m)	0.97 (21 s)
(11, 3)	0.1146	0.86 (10 s)	0.95 (5.2 m)	0.84 (4.3 m)	0.96 (33 s)
(13, 4)	0.2060	0.83 (10 s)	0.93 (8.1 m)	0.78 (36.4 m)	0.95 (36 s)
(15, 5)	0.3135	0.78 (11 s)	0.84 (9.6 m)	0.74 (46.7 m)	0.93 (34 s)
(17, 6)	0.4261	0.70 (13 s)	0.83 (18.6 m)	0.72 (53.6 m)	0.92 (38 s)
(19, 7)	0.5346	0.68 (17 s)	0.75 (24.5 m)	0.70 (1.2 h)	0.90 (40 s)

**Table 4:** The results on (l,d) problem instances with $p_{2d}=0.05$, α=90%.

(l,d)	Time	Predicted Motif	Published Motif
(9, 2)	43 s	TTATCCCTC	TTATCCCTC
(12, 3)	34 s	TTTCCCGTCTGC	CTTTCCCGTCTG
(15, 4)	42 s	GGTTGRAGCTTAGGG	GGTTGGAGCTTAGGG
(18, 5)	38 s	CTTTGCCATATCCATAGG	TTTGCCATATCCATAGGC
(21, 6)	36 s	CAGGTAAACCATATTAAATTA	AGGTAAACCATATTAAATTAC

R: A,G.

**Table 5:** Comparison of (l,d) problem instances with t=600, n=200, and α=90%.

(l,d)	$p_{2d}$	MEME-ChIP	ChIP-Munk	FMotif	AP-ChIP
(9, 2)	0.049	0.96 (12 s)	0.96 (1.8 m)	1.00 (47 s)	1.00 (43 s)
(11, 3)	0.114	0.94 (24 s)	0.92 (2.0 m)	0.99 (7.9 m)	0.98 (46 s)
(13, 4)	0.205	0.90 (38 s)	0.83 (2.4 m)	0.98 (1.45 h)	0.93 (58 s)
(15, 5)	0.319	0.85 (42 s)	0.80 (8.2 m)	–	0.92 (1.1 m)
(17, 6)	0.426	0.80 (45 s)	0.78 (9.6 m)	–	0.89 (1.3 m)
(19, 7)	0.534	0.78 (48 s)	0.76 (10.7 m)	–	0.87 (1.6 m)

**Table 6:** Results on the mESC data set.

Data Set (Seq #)	Time	Predicted Motif	Published Motif
c-Myc (3422)	125 s	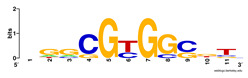	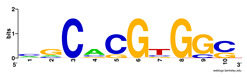
CTCF (39609)	19 s	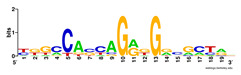	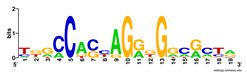
Esrrb (21647)	10 s	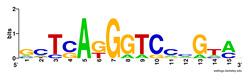	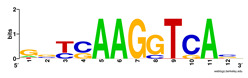
Klf4 (10875)	138 s	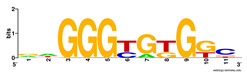	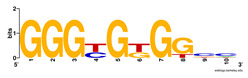
Nanog (10343)	12 s	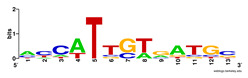	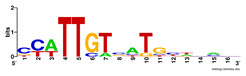
n-Myc (7182)	36 s	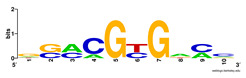	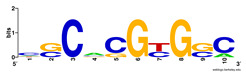
Oct4 (3761)	48 s	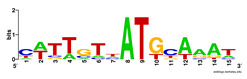	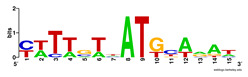
Smad1 (1126)	12 s	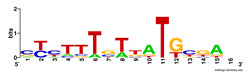	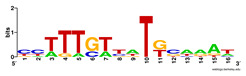
Sox2 (4525)	15 s	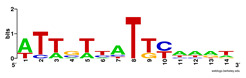	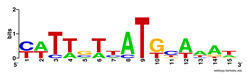
STAT3 (2546)	27 s	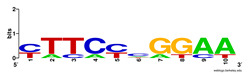	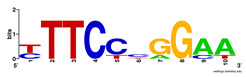
Tcfcp211 (26910)	11 s	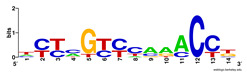	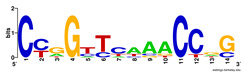
Zfx (10338)	68 s	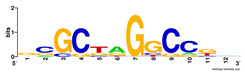	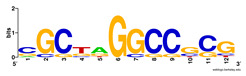

**Table 7:** Results on the ENCODE dataset.

Data Set (Seq #)	AP-ChIP Predicted Motif	MEME-ChIP Predicted Motif	Published Motif
Nfyb (10096)	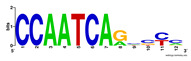	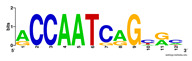	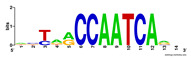
Hnf4 (11045)	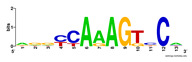	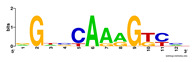	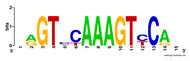
Elf1 (8611)	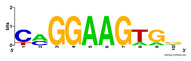	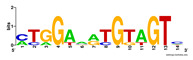	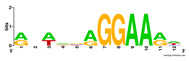
Ets (5525)	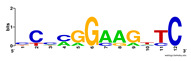	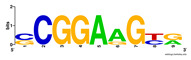	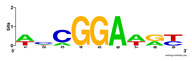
Egr1 (15400)	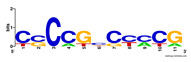	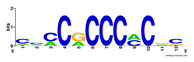	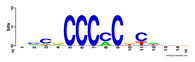
Yy1 (2077)	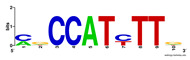	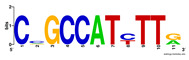	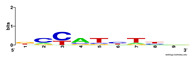
Six5 (4664)	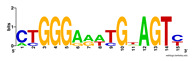	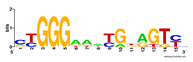	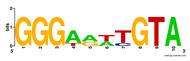
Srf (4903)	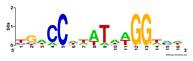	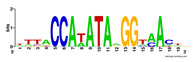	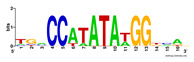
Tal1 (25507)